# A magic pocket: An all-in-one CRISPR toolbox for plants

**DOI:** 10.1093/plcell/koaf120

**Published:** 2025-05-14

**Authors:** Andrea Gomez-Felipe

**Affiliations:** Assistant Features Editor, The Plant Cell, American Society of Plant Biologists; Department of Biology, Indiana University, Bloomington, IN 47405, USA

Imagine you have a magical pocket that holds all the tools you could ever need for crafting the perfect plant. Need to tweak a single gene? Reach in and grab the genome-editing wand. Want to change a base or turn a gene on? No problem, your magical pocket has just the right tool for that. Plant scientists often face the challenge of having the right tools for large-scale applications. Yanhao Cheng and colleagues (Cheng et al. 2025) have addressed this issue by creating a magical pocket for plant scientists, a comprehensive all-in-one CRISPR toolbox that enables genome editing, base editing, and gene activation across a wide range of plant species, including both monocots and dicots (Figure).

**Figure.**
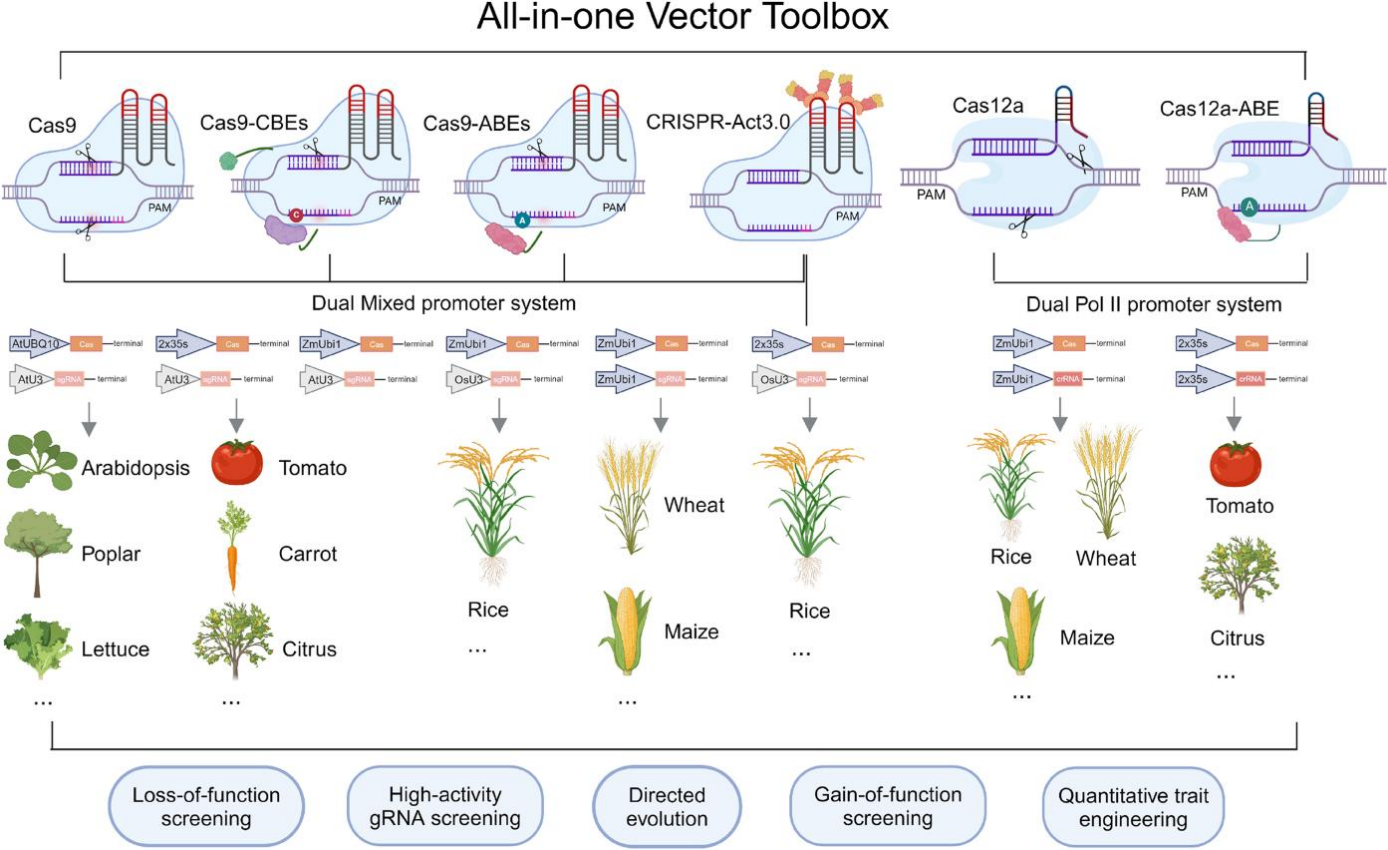
A versatile all-in-one CRISPR vector toolbox for plant genome engineering. This toolkit integrates multiple CRISPR systems—Cas9, Cas12a, base editors (CBEs and ABEs), and CRISPR-Act3.0—under dual promoter systems, enabling efficient genome editing, gene activation, and base editing across a broad range of monocot and dicot species. Applications include loss- and gain-of-function screening, high-activity gRNA screening, directed evolution, and trait engineering. Reprinted from Cheng et al. (2025), Figure 1, panel c.

This magical pocket is packed with 61 all-in-one CRISPR vectors that support a variety of CRISPR-based techniques. This toolkit integrates 1) cutting and editing of genes using Cas9 and Cas12a, 2) the fine-tuning of bases with cytosine and adenine base editing (CBEs and ABEs), and 3) gene activation with CRISPR-Act3.0.

High-throughput, CRISPR-based genetic screening at the genome-wide scale remains a major challenge in plant systems due to the complexity of generating and evaluating large guide RNA (gRNA) libraries (Pan et al. 2023). To address this, Cheng et al. included an approach for gRNA library construction into their all-in-one CRISPR toolkit and made it feasible to conduct locus-specific screens. To demonstrate the versatility of their CRISPR toolbox, they validated its utility in 2 functional genomic screens: herbicide resistance in rice and early flowering induction in *Arabidopsis thaliana*.

In rice, Cheng et al. used the all-in-one base editing system to perform targeted mutagenesis of the *Acetolactate Synthase* gene in rice (*OsALS1*), which encodes a key enzyme in branched-chain amino acid biosynthesis and a common herbicide target. Certain amino acid substitutions create a herbicide-resistant version of the ALS protein (Mazur et al. 1987). They designed 24 CBE and 36 ABE single gRNA (sgRNA) targeting a 324-bp region previously associated with herbicide resistance. Using PAM-less SpRY Cas9 variants, the gRNA libraries achieved complete base coverage of the target region. Editing efficiencies varied across single gRNAs (up to ∼11%), and several amino acid substitutions were recovered. Following bispyribac-sodium selection, 3 resistant plants were identified, carrying either the Y561C (weak resistance) or H541Y (strong resistance) mutations in *OsALS*. Structural analyses suggest these mutations interfere with herbicide binding. These results show the potential of base editing and pooled library screening for direct evolution and trait engineering in plants.

To further validate their system, the authors used the CRISPR-Act3.0 activation system to perform a pooled sgRNA screen targeting the promoter of *FLOWERING LOCUS T* in *Arabidopsis thaliana* (*AtFT*) (Kardailsky et al. 1999). The authors designed a library of 23 sgRNAs spanning the 1-kb upstream region of *AtFT*, whose overexpression is known to trigger early flowering. T1 lines showed early flowering phenotypes, with ∼98% of selected plants displaying strong *AtFT* activation. sgRNA profiling revealed that gR18 was enriched in 22.5% of early-flowering individuals. qPCR analysis of T2 plants carrying gR18 showed a pronounced early flowering phenotype and >50-fold upregulation of *AtFT* expression. This experiment demonstrates the efficacy of the CRISPR toolbox for high-throughput identification of functional activation sgRNAs in planta.

Despite its versatility, challenges remain in delivering CRISPR components to certain plant species, particularly those with lignified secondary cell walls or those requiring regeneration through somatic tissues (Bennur et al. 2025). Nonetheless, the broad applicability of this CRISPR toolbox across both monocots and dicots significantly enhances the ability to manipulate genes and explore complex biological processes at scale.

This work provides a powerful platform for scalable and diverse genome manipulation, bringing plant genome editing closer to the level achieved in mammalian systems. This all-in-one CRISPR toolbox system offers flexible and user-friendly implementation, reducing technical barriers to conducting loss- or gain-of-function at locus level. The magic pocket could facilitate broad applications in plant functional genomics, trait discovery, and crop improvement.

## Recent related articles in *The Plant Cell*

- Dong et al. (2025) developed a fast, automated, scalable, high-throughput pipeline for plant bioengineering through genome editing.
- Lorenzo et al. (2023) presented a gene discovery pipeline that combines multiplex genome editing of whole gene families with crossing schemes to improve complex traits such as yield and drought tolerance, using maize as a model.
